# Impact of varied tillage practices and phosphorus fertilization regimes on wheat yield and grain quality parameters in a five-year corn-wheat rotation system

**DOI:** 10.1038/s41598-024-65784-w

**Published:** 2024-06-26

**Authors:** Hadi Ahmadi, Hossein Mirseyed Hosseini, Farhad Moshiri, Hossein Ali Alikhani, Hassan Etesami

**Affiliations:** 1 Soil Science Department, University of Tehran, Karaj, Iran; 2 Soil and Water Research Institute, Agricultural Research, Education and Extension Organization (AREEO), Karaj, Iran

**Keywords:** Agronomic practices, Baking quality, Phosphorus management, Nutrient content, Tillage methods, Plant sciences, Plant development, Plant physiology, Plant stress responses

## Abstract

Choosing appropriate tillage methods and applying the right amount of chemical fertilizers are pivotal for optimizing wheat management and enhancing wheat quality. This study investigated the influence of conservation agriculture and phosphorus levels on nutrient content, yield components, and quality traits of wheat in a corn-wheat rotation. Conducted over five years in field conditions, the study employed a randomized complete block design with tillage treatments (conventional tillage, CT; minimum tillage, MT; and no tillage, NT) and phosphorus levels (no fertilizer use, P0; and 100% fertilizer recommendation, PR) as factors. Soil samples were collected during the fourth year (2021–2022). Results revealed significant impacts of tillage methods and phosphorus levels on wheat straw and grain nutrient composition, yield components, and quality traits. Conventional tillage yielded the highest values for protein content (12%), Zeleny sedimentation volume (20.33 mL), hardness index (45), water absorption (64.12%), and wet gluten content (25.83%). Additionally, phosphorus fertilizer application positively influenced protein percentage, gluten weight, and gluten index. The study highlights the potential of strategic soil management, particularly conventional tillage combined with phosphorus fertilization, to enhance wheat quality and yield. By elucidating these relationships, the findings contribute to optimizing wheat cultivation practices and advancing the development of superior wheat cultivars for baking applications.

## Introduction

Wheat (*Triticum aestivum* L.) is a crucial staple in human nutrition and a significant agricultural product worldwide. Rising global population and increasing wheat prices emphasize the need for self-sufficiency in this strategic commodity. Choosing appropriate tillage methods and applying the right amount of chemical fertilizers are crucial for achieving optimal efficiency in crop management and quality improvement^1^. The intricate interplay between agronomic practices and environmental factors profoundly shapes the yield and quality of wheat grain^2–4^. Achieving optimal wheat yields faces challenges such as unfavorable soil characteristics, light textured and acidic soils, as well as inadequate rainfall during pivotal growth stages, leading to yield reduction^5,6^. Another critical factor impacting yield and quality is the sequence of crops in a rotation, with the most favorable yields often seen when wheat follows leguminous crops^7,8^. In a previous study, higher levels of NPK fertilization, especially in rotations where the proportion of corn or wheat is 50% or higher, led to significantly higher yields and better nutritional quality^9^. In other studies, wheat-corn rotation improved yield components such as protein content, plant height, and grain yield^10,11^. The inclusion of winter wheat in a no-tillage corn-soybean rotation (corn-soybean-wheat) also led to increased soybean yields in some years but decreased corn yields compared to a corn-soybean rotation^12^. Mourtzinis, et al.^13^ found that yearly crop rotation of corn and soybean increased corn grain yields by 15–18% and soybean yields by 24–31% compared to continuous cropping. However, conventional practices often entail planting wheat after other cereals, thereby promoting weed proliferation^14,15^ and susceptibility to diverse diseases^16^. These combined factors frequently contribute to a noticeable reduction in grain yield and quality, characterized by decreased grain density and uniformity, and an elevated content of grain ash^17,18^. Tillage practices also wield significant influence over both the yield and quality of wheat grains, predominantly by altering the physical, chemical, and biological attributes of the soil, which subsequently impact plant growth^19,20^. Tillage is aimed at optimizing conditions for robust grain production, yet opinions on the most effective tillage methods remain diverse and depend on variables like climate and cultivated crops^14,21,22^. Notably, plant performance is a complex outcome of various climatic and agronomic factors, with studies highlighting higher yields in no-tillage treatments in regions with limited rainfall^23,24^. While tillage methods play a role in shaping wheat grain quality, weather conditions and cultivar traits often exert more influence than tillage practices^25,26^.

Studies indicate that tillage practices can influence the efficacy of fertilizers and the overall soil quality, thereby impacting crop yield^1^. Tillage and chemical fertilizers show a significant correlation^27^. In a prior investigation, a progressive rise in tillage operations led to increased soil bulk density, resulting in diminished nitrogen, potassium and phosphorus uptake and ultimately affecting the quality of corn and wheat grain^28,29^. Phosphorus is vital for wheat nutrition and enhancing grain quality. The interplay between external and internal phosphorus sources significantly influences grain phosphorus loading in wheat. High phosphorus supply promotes substantial P uptake and optimized P remobilization to the grains in wheat plants^30^.

The exploration of diverse soil tillage methods and phosphorus fertilization in the context of wheat cultivation is crucial for optimizing resource utilization, improving grain quality, and mitigating environmental impact. As global agriculture faces increasing challenges from climate change and resource limitations, the identification of efficient and sustainable practices becomes paramount. With these intricate dynamics in mind, the present study seeks to delve into the quantitative attributes and quality aspects of winter wheat, dissecting how different tillage practices—conventional tillage (CT), reduced tillage (RT), and no tillage (NT)—coupled with the application or absence of phosphorus fertilizer, collectively influence these factors. Our aim was to provide a comprehensive understanding of how these elements synergistically impact the concentration of crucial nutritional components (N, P, and K), overall yield, yield components, and the multifaceted quality traits of wheat in a five-year corn-wheat rotation system.

## Materials and methods

### Study area and experimental design

In this assay, no permissions or licenses are needed to collect plant materials, and all procedures were conducted in accordance with the guidelines.

The present research was conducted in 2016–2022 at the research farm of the Water and Soil Institute (located at 35°45΄3.43˝ N, 50°57΄14.53˝ E, and 1256 m above sea level) in Karaj, Iran. The study area experiences an annual rainfall of approximately 400 mm, with maximum and minimum temperatures reaching 27.9°C and 2.7°C, respectively, and an average annual temperature of 15.8°C. Soil chemical properties including pH^31^, electrical conductivity (EC) ^32^, organic matter^33^, total nitrogen^34^, available phosphorus^35^, and available potassium^31^ were measured. The soil classification is designated as: Loamy skeletal, mixed, mesic Typical Haploxerepts. The properties of farm soil are shown in Tables 1 and 2.

**Table 1:** The properties of the soil before the last cultivation of wheat or the start of the experiment.

Treatmeants	TN (%)	Available-P (mg Kg^−1^)	Available-K (mg Kg^−1^)	OC (%)	B.D (g cm^−3^)	EC (dS m^−1^)	pH
NT-P0	0.073	6.08	211.16	0.79	1.95	0.88	8.23
NT-PR	0.076	18.00	201.36	0.80	1.9	0.94	8.22
MT-P0	0.06	7.14	275.60	0.70	1.89	0.94	8.06
MT-PR	0.087	23.18	277.45	0.83	1.93	1.19	8.01
CT-P0	0.073	7.40	250.88	0.72	1.84	0.91	8.01
CT-PR	0.074	17.20	279.38	0.75	1.79	0.79	7.99

*CT* conventional tillage, *MT* minimum tillage, *NT* no tillage, *P0* no phosphorus fertilizer use, *PR* 100% fertilizer recommendation, *TN* total nitrogen, *OC* organic matter, *B.D* bulk density, and *EC* electrical conductivity.

**Table 2:** Soil characteristics before implementing the plan in the year 2016 (0–20 cm).

TN (%)	Available-P (mg Kg^−1^)	Available-K (mg Kg^−1^)	OC (%)	B.D (g cm^−3^)	EC (dS m^−1^)	pH	SP (%)	Clay (%)	Silt (%)	Sand (%)	TNV (%)
0.06	12.2	243	0.64	1.50	1.08	7.46	32.5	28	32	40	10.5

*TN* total nitrogen, *OC* organic matter, *B.D* bulk density, *EC* electrical conductivity, *SP* saturation percentage, and *TNV* total neutralizing value.

**Figure 1:**
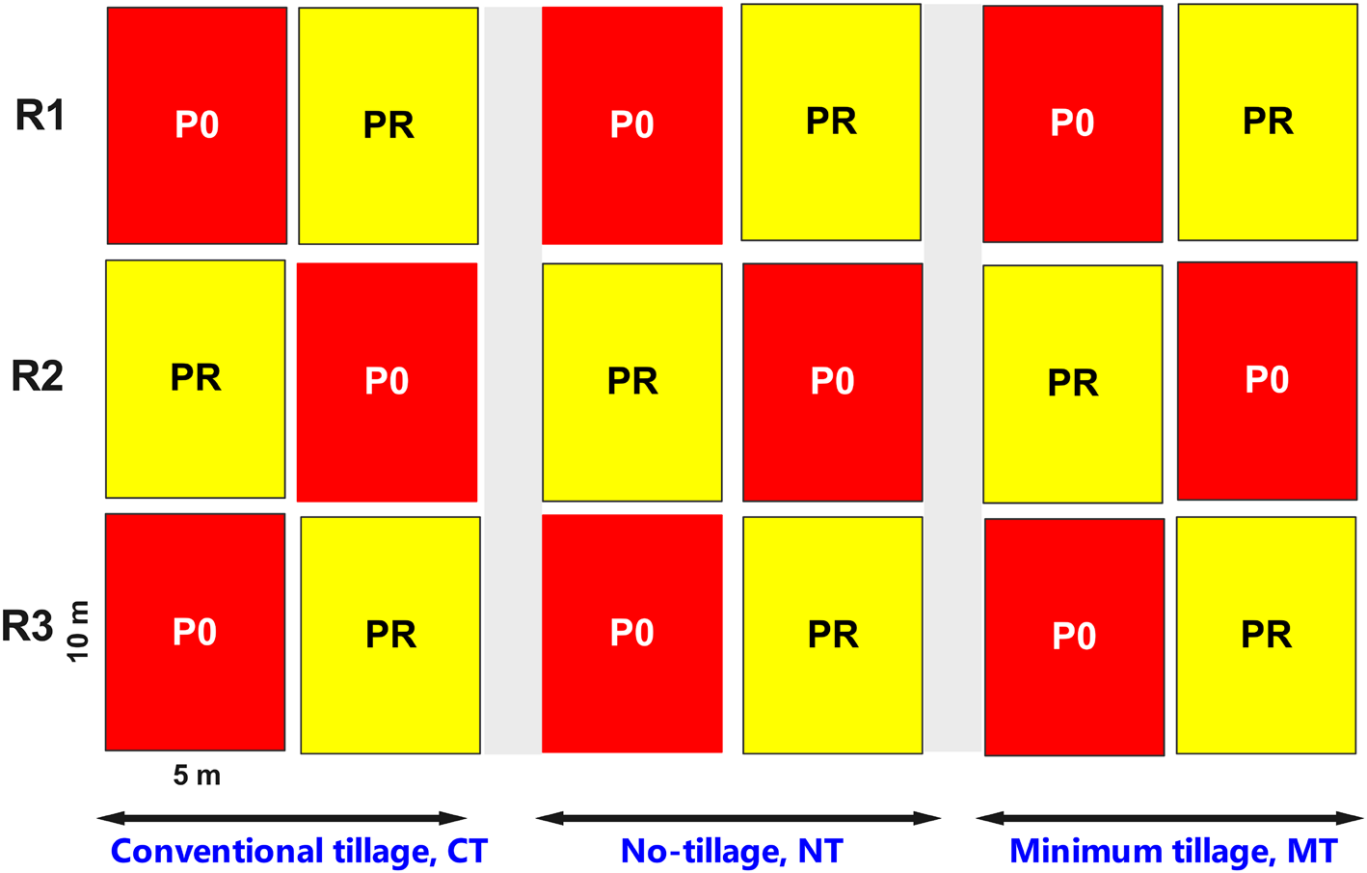
The split-plot design layout utilized in the study. The main plots were allocated to different tillage methods at three levels: Conventional tillage (CT), no-tillage (NT), and minimum tillage (MT). Subplot factors were assigned to phosphorus (P) treatments at two levels: No P consumption (P0) and 100% P fertilizer recommendation (PR) based on soil test results.

The experiment was conducted within a fixed plot of land that has been subjected to varying tillage and residue preservation methods since the autumn of 2016, with a crop rotation of wheat and corn. For this study, a randomized complete block design with three replications was utilized in the form of split plots (Fig. 1). The experimental treatments consisted of: (***a***) different tillage methods at three levels as main plots, namely conventional tillage, CT (consisting of reversible plowing, two rounds of crossed disc, and planting with seed sowing machine, fertilizer drill, and grain drill), no-tillage, NT (comprising direct sowing with seed drill, fertilizer drill, grain drill, and without plowing), and minimum tillage, MT (involving two rounds of crossed disc and planting with seed drill, fertilizer drill, and grain drill); and (***b***) phosphorus treatments at two levels as subplot factor, including no phosphorus consumption (P0) and 100% phosphorus fertilizer recommendation (PR) based on soil test.

Each sub-plot measured 50 square meters in area. In the conventional tillage plot, all crop residues were removed. However, in the minimum-tillage and no-tillage plots, only standing crop residues (approximately thirty percent) were retained, while the remaining residues were removed from the soil. Phosphorus was applied during planting in strip form using triple superphosphate as the source. Other key nutritional elements such as potassium, nitrogen, and trace elements were uniformly applied throughout the project based on soil test results and expected grain yield. The recommended phosphorus fertilizer application rate was 150 kg ha^−1^ of triple superphosphate. For urea fertilizer, a total of 150 kg ha^−1^ was recommended, to be applied in three installments at the stages of planting, tillering or leafing, and flowering or crowning. Additionally, 150 kg ha^−1^ of potassium sulfate was recommended for optimal plant growth. Irrigation was carried out using a tape method. In this assay, the growth scale used was Zadoks. In this study, Pishgam wheat variety was utilized at a density of 200 kg per hectare, with planting taking place on November 20th and harvesting on June 20th. Additionally, fodder corn (single cross-704 variety) was used at a planting density of 25 kg per hectare, with planting occurring on July 14th and harvesting on October 7th over the same 5-year timeframe.

### Measurements

To capture the quantitative characteristics of wheat within each treatment, a metal box measuring one square meter was placed randomly, and all spikes contained within the box were meticulously harvested in triplicate. These spikes were then counted and weighed. Following this, the separation of grain from straw ensued, with individual weights recorded. The cumulative yield per hectare was subsequently computed. Calculating the weight of one thousand seeds necessitated counting one hundred seeds, which were then multiplied by ten. For gauging spike length, plant height, and the number of grains per spike, ten spikes were meticulously collected from each treatment. The spike lengths' length and the overall plant height were measured using a ruler. Additionally, the grains within these spikes were counted and subsequently divided by ten to achieve accurate counts. The concentration of potassium, phosphorus and nitrogen of wheat straw and grain was measured and reported according to the common standard method. Total phosphorus and potassium in wheat grain and straw were measured using the association of Official Analytical Chemists (AOAC) 999.10 method. Initially, 0.5 g of the ground sample was weighed into Teflon tubes designed for a microwave device. To this, 5 mL of concentrated nitric acid (with 65% high purity) and 2 ml of 30% hydrogen peroxide were added. The tubes were then inserted into the microwave for digestion. Finally, the concentration of elements was determined using an inductively coupled plasma atomic emission spectrometer (ICP-OES) following the AOAC 999.10 protocol. The total nitrogen concentration in the samples was determined using the Kjeldahl method. Initially, plant extracts were obtained through a process involving the digestion of dried samples with concentrated sulphuric acid followed by perchloric acid. To start, 0.5 g of oven-dried ground sample was placed in a 150 mL Kjeldahl flask, to which 0.5 mL of concentrated H_2_SO_4_ was added, allowing it to sit overnight. Subsequently, 2.5 mL of perchloric acid was introduced into the flask. The flasks were then gradually heated until reaching a temperature of 200 °C, continuing until the digest became clear and colorless. After cooling, the content was transferred into a 100 mL volumetric flask, and the volume was adjusted with distilled water. A reagent blank was also prepared using a similar procedure. This digestion process was specifically designed for nitrogen determination. Nitrogen in the digest was subsequently estimated by distillation with 40% NaOH, followed by titration of the trapped distillate in H_3_BO_3_ with 0.01 N H_2_SO_4_, following the method outlined by Page et al.^36^.

Upon harvesting, 500 g of flour from each treatment underwent thorough qualitative testing at the Grain Chemistry Laboratory, Seedling and Seed Breeding Research Institute, Karaj, Iran. The analysis encompassed: grain protein percentage, grain hardness percentage, flour's water absorption percentage, and bread volume. These parameters were determined in accordance with the methodology outlined by Norris^37^. Furthermore, employing a gluten washing machine in conjunction with a centrifuge, the quantity of wet gluten was determined, adhering to the standards outlined in the International Cereal Association's Standard No. 137.

### Statistical analysis of data

Once the collected data was tested for homoscedasticity (using the Levene test) and normality (using the Kolmogorov–Smirnov test), they were subjected to statistical analysis using a two-way analysis of variance in SAS v.9.1 (SAS Institute Inc., Cary, NC). To estimate significant differences among treatments, Duncan's multiple range test (*p* ≤ 0.05) was used to calculate means and standard errors. Additionally, Pearson's two-tailed test (*p* ≤ 0.05) was employed to investigate the relationship among qualitative characteristics of wheat plant**.**

## Results

### Impact of treatments on N, P, and K concentration of straw and grain

The individual impact of tillage on nutrients (N, P, and K) in wheat straw and grain, except for N percentage in wheat straw, was found to be significant (Table S1). Moreover, the sole effect of fertilization on N content in straw and wheat grain was deemed insignificant. However, it had a notable impact on the K and P percentages in both straw and grain, with significance levels at 1% and 5%, respectively. Regarding the interaction between tillage and fertilization, it was not significant, except for grain N concentration (*p* < 0.05), as well as straw K and P concentration (*p* < 0.01), among other nutritional elements in wheat straw and grain (Table S1).

**Figure 2:**
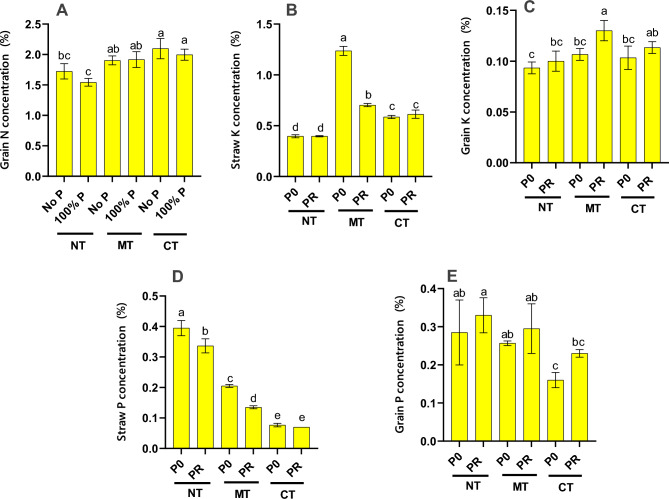
Effect of various treatments on wheat grain N concentration (**A**), wheat straw K concentration (**B**), wheat grain K concentration (**C**), wheat straw P concentration (**D**), and wheat grain P concentration (**E**) in a five-year corn-wheat rotation. CT, conventional tillage; MT, minimum tillage; NT, no tillage; P0, no phosphorus fertilizer use; and PR, 100% fertilizer recommendation. Different letters indicate significant differences according to the Duncan at *p* ≤ 0.05.

The conventional tillage treatment without P fertilizer exhibited the highest wheat grain N content at 2.10%. However, this measurement did not demonstrate a statistically significant difference when compared to the CT-P0, MT-P0, and MT-PR treatments. Conversely, the lowest N content was recorded in the NT-PR treatment (Fig. 2A). The maximum K content in straw was observed in the minimum tillage treatment without the application of P fertilizer (1.24%), followed by the minimum tillage treatment with P fertilizer (0.70%) (Fig. 2B). Conversely, the lowest K content in straw was recorded in the no-tillage treatments, both with and without P fertilizer application, at 0.40% (Fig. 2B). Regarding K content in wheat grain, the highest concentration was found in the conventional tillage treatment (0.118%), although it did not exhibit a statistically significant difference from the minimum tillage treatment (0.108%). Conversely, the lowest K content in wheat grains was observed in the no-tillage treatment at 0.097% (Fig. 2C). The application of P fertilizer significantly impacted the K percentage of wheat seeds, leading to a notable increase (1.1 times) in K concentration (Table S3). Consequently, the highest K percentage in wheat grain among the minimum tillage treatments was observed when P fertilizer was utilized (0.13%), although it did not exhibit a significant difference compared to other treatments (Fig. 2C). Regarding straw P, the highest percentage was recorded in the no-tillage treatment without P fertilizer application (0.40%), while the lowest amount was observed in the conventional tillage treatment with P fertilizer (0.07%) (Fig. 2D). Similarly, for wheat grain P, the highest percentage was observed in the no-tillage treatment with P fertilizer (0.33%), contrasting with the lowest amount in the conventional tillage treatment without P fertilizer (0.16%) (Fig. 2E). These results suggest that the minimum tillage system without P fertilizer and the no-tillage system with P fertilizer may be the most beneficial management practices for optimizing nutrient content in wheat under the conditions of this study.

### Impact of treatments on yield and its components

**Figure 3:**
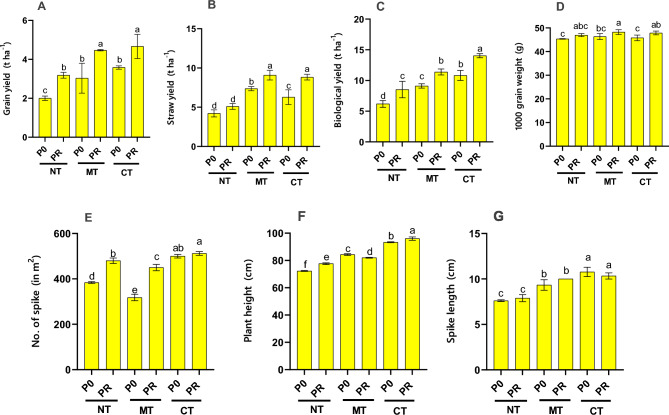
Effect of various treatments on wheat grain yield (**A**), wheat straw yield (**B**), wheat biological yield (**C**), wheat 1000 grain weight (**D**), No. of spike (**E**), plant height (**F**), and spike length (**G**) in a five-year corn-wheat rotation. CT, conventional tillage; MT, minimum tillage; NT, no tillage; P0, no phosphorus fertilizer use; PR, 100% fertilizer recommendation. Different letters indicate significant differences according to the Duncan at *p* ≤ 0.05.

The individual impact of tillage treatment proved significant across all yield parameters and wheat yield components, except for the weight of 1000 wheat grains (Tables S1, S2, and S4). Similarly, fertilization exerted a significant influence on all parameters, barring the weight of 1000 grains and the length of the spike, with a probability level of 1%. Notably, the interaction effect between tillage and fertilization was only insignificant concerning spike length, while it demonstrated significance in all other parameters at the probability level of 1%. The conventional tillage treatment with P fertilizer consumption exhibited the highest wheat grain yield (4.67 tons/ha), closely followed by the no-tillage treatment with P fertilizer consumption (4.47 tons/ha), with no significant difference between them. Conversely, the lowest gain yield was recorded in the no-tillage treatment without P fertilizer (2.00 tons/ha) (Fig. 3A). Across all tillage methods, the utilization of P fertilizer led to an increase in wheat grain yield. Additionally, the maximum yield of wheat straw was observed in the minimum tillage treatment with P fertilizer application (9.08 tons/ha), although it did not significantly differ from the conventional tillage treatment with P fertilizer (8.83 tons/ha). In contrast, the lowest wheat straw yield occurred in the no-tillage treatment without P fertilizer (4.22 tons/ha) (Fig. 3B). As depicted in Fig. 3B, across all tillage methods, the application of P fertilizer significantly enhanced wheat straw yield.

The highest and lowest biological yields were recorded in the conventional tillage treatment with P fertilizer (14.03 tons/ha) and the no-tillage treatment without P fertilizer (6.18 tons/ha), respectively (Fig. 3C). Moreover, Fig. 3C illustrates that P fertilizer application consistently increased the biological yield of wheat across all tillage methods. Furthermore, the weight of 1000 grains of wheat showed variation, with the highest observed in the minimum tillage treatment with P fertilizer application (48.23 g), while the lowest was in the no-tillage treatment without P fertilizer (45.37 g) (Fig. 3D). Notably, P fertilizer application contributed to an increase in the weight of a thousand wheat grains across all tillage treatments. Additionally, the number of spikes per square meter was highest in the conventional tillage treatment with phosphorus fertilizer (512), showcasing a notable increment compared to the treatment without phosphorus fertilizer (318) (Fig. 3E). This suggests that P fertilizer treatments effectively boost the number of wheat spikes. Furthermore, the height of the wheat plant increased with P fertilizer application across all tillage treatments, with the tallest plants observed in the conventional tillage treatment with P fertilizer (96 cm) (Fig. 3F). The longest spike length was also observed in the conventional tillage treatment without P fertilizer (10.78 cm), underlining the role of P fertilizer in enhancing wheat spike length (Fig. 3G). The results suggest that the conventional tillage system combined with P fertilizer application may be the most optimal management practice to achieve the highest wheat grain and straw yields. However, the no-tillage system with P fertilizer also showed promising results in terms of maintaining high grain and straw yields.

### Impact of treatments on quality traits of wheat grains

The impact of tillage practices on grain protein percentage, gluten moisture percentage, and gluten index reached statistical significance at the 1% level, while Zeleny sedimentation volume, bread volume, hardness index, and flour water absorption were significant at the 5% level, with no significance observed on grain moisture content (Table S5). Similarly, P fertilization significantly affected only gluten content and gluten index (*p* < 0.01), with no significant impact on other parameters. Examination of the interaction effect between tillage and fertilization revealed that bread volume and flour water absorption were influenced by flour gluten content and gluten index (Table S5).

Under the sole influence of tillage, grain protein percentage exhibited significance, while the effects of fertilization alone and the interaction between tillage and fertilization were not statistically significant (Table S6). Consequently, the highest grain protein percentage was recorded in the conventional tillage treatment (12%), whereas the lowest was observed in the no-tillage treatment (11.15%), with no notable difference between minimal tillage and no-tillage treatments. Neither grain moisture percentage nor flour water absorption was influenced by the individual effects of tillage or fertilization, nor by their interaction. Moreover, the impact of fertilization alone and the interaction between tillage and fertilization on Zeleny sedimentation volume were insignificant, with only the effect of tillage showing significance (Table S5).

**Figure 4:**
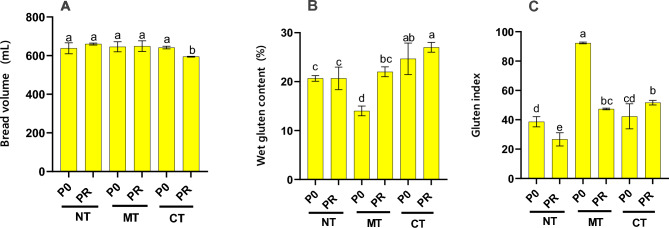
Effect of various treatments on wheat bread volume (**A**), wet gluten (**B**), and gluten index (**C**) in a five-year corn-wheat rotation. CT, conventional tillage; MT, minimum tillage; NT, no tillage; P0, no phosphorus fertilizer use; PR, 100% fertilizer recommendation. Different letters indicate significant differences according to the Duncan at *p* ≤ 0.05.

The Zeleny sedimentation volume reached its peak in the conventional tillage treatment (20.33 mL), showing no significant difference from minimal tillage (19.17 mL), yet notably exceeding the volume under no-tillage conditions (18.33 mL) (Table S6). Additionally, the interaction effect of tillage and fertilization significantly influenced bread volume (*p* < 0.05). Consequently, the highest bread volume was observed in the no-till treatment with P fertilizer (660 mL), comparable to other treatments except for conventional tillage with P fertilizer (594.67 mL) (Fig. 4A). Grain hardness index was solely affected by tillage, with the highest value recorded in conventional tillage (45), though not significantly different from no-tillage (Table S6). Furthermore, the interaction effect of tillage and fertilization significantly impacted gluten parameters. The highest gluten content was under conventional tillage with P fertilizer (27%), while the lowest was in tillage without phosphorus fertilizer (14%) (Fig. 4B). Similarly, the interaction effect of tillage and fertilization significantly influenced the gluten index. The highest index was under tillage without P fertilizer (92.33), contrasting with the lowest observed in no tillage treatment with P fertilizer (26.67) (Fig. 4C). Comparing averages revealed that P fertilizer use increased protein percentage (by 0.9%) and gluten content (by 3.44%), while decreasing the gluten index. Except for bread volume, other parameters showed slight increases due to P fertilizer use. In summary, conventional tillage treatments with P fertilizer achieved the highest bread volume and flour water absorption, whereas the least tillage treatment without P fertilizer yielded the highest gluten index.

## Discussion

Choosing the right tillage methods and applying the appropriate amount of chemical fertilizers are essential for optimizing wheat management and enhancing the quality of this plant. In this study, the nutrient levels in both wheat straw and grain were influenced by tillage methods and the application of P fertilizer, excluding straw nitrogen. Notably, the P concentration in wheat straw and grain was higher under the no-tillage treatment compared to other cultivation methods. The decline in the P percentage in wheat grain in conventional tillage treatments, in contrast to conservation tillage, is likely attributed to the higher grain yield in crops grown under conventional tillage. This suggests a dilution effect on the P concentration in wheat grain due to the increased overall grain production in the conventional tillage system. In a long-term study conducted by Campbell, et al.^38^, the impact of plow management, fertilizer application, and crop rotation on winter wheat nutrient concentrations was investigated. Their findings revealed that the concentration of P in grain from fallow-wheat treatments was lower than in corresponding treatments with stubble-wheat. This decrease in grain P concentration was attributed to the higher yield in the fallow crop, leading to a dilution effect on the concentration of plant elements. Similarly, in a study by Zhang, et al.^39^ focusing on crop yield, P uptake, and organic P fractions in a rice-canola rotation in central China, it was reported that the no-tillage method resulted in increased P uptake in rice grain and straw as well as canola, compared to conventional tillage. Nitrogen and K levels in wheat straw and grain under no-tillage were lower than those in minimal tillage and conventional tillage. The surface retention of plant residues in the no-tillage treatment slowed down decomposition and mineralization, leading to reduced nutrient availability in the soil and subsequently lower nutrient concentrations in the plants. Studies by Loke, et al.^40^ and Sarker, et al.^41^ highlighted that the higher mineralization of soil organic carbon under conventional tillage and reduced tillage compared to no-tillage resulted in lower net N and K availability. The use of P fertilizer in the soil increased wheat yield, leading to elevated nutrient extraction from the soil. However, despite increased nutrient extraction, the availability of these elements remained constant, resulting in a dilution effect in the plant and reduced nutrient concentrations. Contrary to the general trend, P supplementation did not lead to a dilution effect in straw and seeds, as the increased availability of P prevented P deficiency. McBeath, et al.^42^ additionally noted a synergistic relationship between soil moisture and P nutrition for plants, emphasizing that P fertilizer application enhances the plant's utilization of soil P.

In this study, wheat yield and its components exhibited a pattern of conventional tillage > minimum tillage > no-tillage. Tillage practices play a significant role in shaping the growth and differentiation of yield structure elements, ultimately influencing grain yield^43^. Several studies have consistently reported reduced wheat yield under conservation tillage when compared to conventional tillage^44–46^. Rieger, et al.^47^ found that the reduction in grain yield due to reduced tillage was associated with decreased grain density on the cob in corn and 1000-grain weight in wheat.

The lower yield and yield components observed under no-tillage treatments compared to conventional tillage may be attributed to cooler and wetter soil conditions in early spring, potentially leading to delayed emergence and reduced yield^48,49^. Other factors contributing to reduced yields under no-tillage include the presence of weeds^50^, the accumulation of plant residues from the previous crop disrupting seed germination, and hindered root growth due to increased soil density under conservation tillage^51^. Possible reasons for decreased wheat yield in this study include competition with numerous weeds for nutrients and water, the presence of corn residues on the soil surface from the previous crop, and dense corn roots impeding seed germination and subsequent growth. Furthermore, the rise in soil pH in no-tillage treatments (8.22–823), as opposed to other tillage methods (Table 1), may impact wheat yield reduction due to its influence on nutrient availability, particularly phosphorus. Soil pH plays a crucial role in the availability of P to plants^52^. The application of P fertilizers can also affect the availability of P in the soil, depending on the soil pH and the type of P fertilizer used^53^. These factors, along with a relative decrease in the weight of a thousand wheat grains and reduced spike length, contribute to a lower grain yield and overall biological performance under no-tillage. The application of P fertilizer had a positive impact on both yield and yield components, leading to an increase in all wheat characteristics. This aligns with findings from various studies demonstrating an enhanced wheat yield and its components with phosphorus fertilizer application^54–56^. Phosphorus is an essential element for plants, and its increased application results in higher concentrations in both grain and straw^57,58^. The use of P fertilizer also leads to an increase in the weight of 1000 seeds, contributing to higher seed yield, and enhances grain filling^59^.

In this study, the weight of 1000 grains remained unaffected by both tillage management and the application of P fertilizer. Interestingly, this contrasts with numerous prior studies that consistently reported higher 1000-grain weights in conventional tillage compared to no-tillage and minimum tillage systems^60–64^. A review of experiments in Sweden by Arvidsson, et al.^65^ also documented an increase in grain yield with greater tillage intensity. Seepamore, et al.^44^ suggested that the presence of plant residues on the soil surface in no-till conditions might reduce grain yield. This could be attributed to delayed germination and establishment, especially under dry conditions where poor soil-seed contact occurs^65,66^. Additionally, studies have reported soil organic carbon accumulation up to a depth of 10 cm in no-tillage compared to conventional tillage, with less decomposition and mineralization of organic carbon in no-till systems affecting nutrient availability^40,67,68^. In semi-arid climates on sandy soil, Sarker, et al.^41^ found that soil organic carbon mineralization and the availability of N, P, and sulfur were significantly lower under conventional tillage and reduced tillage compared to no-tillage. The higher intensity of N mineralization with increased tillage intensity has been proposed as a reason for the elevated levels of 1000-grain weight in some studies^60,62,63^. However, conflicting results also exist. Rashidi, et al.^69^ indicated that different tillage treatments did not affect the yield and quality characteristics of wheat. In light of these varied findings, it appears that the short-term impact of tillage on yield and yield components may not be significant across all studies.

Whole wheat is a valuable source of energy and essential food components, with protein being a crucial element^70,71^. The protein content in wheat grains and flour typically falls within the range of 7% to 22%, with a predominant concentration between 10 and 15%^72^. Genetic factors, encompassing species and diversity, contribute to approximately one third of this variation, while the remaining two-thirds are influenced by environmental factors, including climate conditions, soil composition, atmospheric CO_2_ concentration, and various crop management practices such as fertilization, tillage, seeding, irrigation, and crop rotation. The interaction among these factors further shapes the protein content^73,74^. For bread-making, the flour should ideally contain more than 10% protein, equating to over 8% gluten protein, as protein content significantly determines the ultimate quality of the end product. A sufficient quantity of gluten protein is essential for establishing a continuous protein network in the dough, stabilizing gas bubbles produced by yeast, and forming pore walls in baked goods^71^. In your study, it was observed that the impact of tillage treatments on wheat quality characteristics outweighed the effect of fertilization. The wheat grain protein percentage exhibited the pattern of conventional tillage > minimum tillage > no-tillage, ranging from 10.80% to 12.30%. Higher protein content in flour has been associated with increased dough elasticity, and approximately 20% of changes in bread quality attributes can be attributed to protein levels^75^. According to the findings reported by Fowler and Delaroche^76^, protein content serves as a crucial variable in predicting bakery quality. The impact of protein quantity on quality properties, such as the valorimetric number of bread volume and the Farinograph resistance index (measuring the stability of dough versus its tendency to loosen), deserves careful consideration^76,77^. Buczek, et al.^78^ observed significantly higher protein content in grain, along with gluten, sedimentation index, grain number, and flour gluten index in CT treatment compared to no-tillage. Contrary to these findings, studies by Woźniak and Gos^79^ and Taner, et al.^80^ did not reveal significant differences in the early quality characteristics of wheat based on tillage treatments. Šíp, et al.^63^ suggested that CT treatment, as opposed to reduced tillage and no-tillage, leads to a more efficient use of nitrogen, influencing the growth and particularly the protein content in wheat grain. Kerbouai, et al.^81^ associated this with increased organic matter decomposition and extended root penetration in the soil under conventional tillage. Jaskulska, et al.^82^ demonstrated a reduction in ash content in flour from CT treatment compared to RT and NT. However, according to Woźniak and Rachoń^46^, the combined influence of wheat cultivar genetic traits, environmental conditions, and their interaction has a more pronounced effect on the qualitative characteristics of wheat grain and flour than tillage treatments. Research by Liniņa and Ruža^83^ highlighted that wheat quality parameters, including grain filling, significantly depend on weather conditions, grain storage period, and the applied nitrogen dose. Hofmeijer, et al.^84^ proposed that lower soil compaction in CT treatment, in comparison to RT and NT, may lead to increased nutrient uptake, including nitrogen, by wheat, thereby enhancing grain quality. This effect is particularly notable under climatic conditions conducive to the mineralization of post-harvest residues in RT and NT.

The protein content in wheat grain exhibits variation across different parts of the grain, with the middle part of the endosperm often having a lower protein content than the outer part. The predominant type of protein in wheat grain is gluten^85^. Gluten possesses the remarkable ability to absorb 2 to 3 times its own weight in water, forming the structure of the dough and providing it with stretchability. The SDS precipitation test serves as an indicator to assess gluten quality, and it exhibits a strong correlation with other traits associated with gluten strength^85^. Consistent with these characteristics, several studies have reported a significant decrease in grain protein content under conservation tillage treatments such as reduced tillage and no-tillage^24,86–88^. In contrast, López-Bellido, et al.^60^ reported higher protein content under conventional tillage than no-tillage. Additionally, Pringas and Koch^89^ found that protein content and sediment value were significantly reduced in the no-tillage treatment compared to tillage-based treatments.

The Zeleny sedimentation volume was influenced by tillage practices, with the lowest value observed in the no-tillage treatment. However, P fertilizer consumption did not have a significant impact on this parameter. Zeleny sedimentation volume, often referred to as sedimentation value, measures the degree of sedimentation of flour suspended in a lactic acid solution over a standard time interval, serving as an indicator of baking characteristics. Mirza Alizadeh, et al.^90^ noted a direct relationship between the volume of zinc sediment and the levels of wet protein and gluten. In this study, the Zeleny sedimentation volume demonstrated the highest correlation with protein percentage, as outlined in Table 3. A higher quality of gluten and increased gluten content typically leads to slower sedimentation and higher Zeleny number values^91^. This underscores the importance of gluten characteristics, including quality and quantity, in influencing sedimentation volumes and, consequently, baking attributes.

**Table 3:** Pearson correlation among quality traits of wheat grains.

	Protein content (%)	Zeleny sedimentation volume (mL)	Bread volume (mL)	Moisture (%)	Hardness index	Water absorption (%)	Gluten weight	Gluten index
Protein content (%)	1							
Zeleny sedimentation volume (mL)	0.665**	1						
Bread volume (mL)	− 0.584**	− 0.620**	1					
Moisture (%)	− 0.073^ ns^	0.393^ ns^	0.117^ ns^	1				
Hardness index	0.378^ ns^	0.584*	− 0.695**	0.037^ ns^	1			
Water absorption (%)	0.533*	0.181^ ns^	− 0.789**	− 0.541*	0.591**	1		
Gluten weight	0.580**	0.398^ ns^	− 0.455*	− 0.154^ ns^	0.603**	0.603**	1	
Gluten index	0.254^ ns^	0.068^ ns^	− 0.108^ ns^	0.112^ ns^	− 0.387^ ns^	− 0.085^ ns^	− 0.549*	1

Respectively; ns, not significant; *significant at *p* < 0.05; **significant at *p* < 0.01.

The percentage of gluten exhibited an increase with the intensification of tillage practices and P fertilizer consumption. Surprisingly, the results indicated that a higher amount of wet gluten does not necessarily indicate greater gluten strength. Wet gluten content serves as a quantitative measure of gluten-forming proteins in wheat flour, primarily influencing dough mixing and baking properties^92^. Oručević-Žuljević, et al.^93^ suggested that a minimum of 25% wet gluten content is necessary for ensuring good baking quality in wheat products. In this study, comparable wet gluten content was achieved only in the conventional tillage treatment, irrespective of P fertilizer application. Kerbouai, et al.^81^ demonstrated that soil management significantly influenced grain quality in terms of protein content and wet gluten under conservation agriculture, achieving 11.15% for protein content and 17.68% for wet gluten. In contrast, conventional tillage resulted in 11.92% for protein content and 18.75% for wet gluten, aligning with the findings of the current study. Colecchia, et al.^86^ also observed a lower percentage of wet gluten and wheat protein in no-till conditions compared to other tillage treatments. Woźniak^94^ supported the idea that reduced tillage contributes to lower wet gluten and protein content, along with a reduced grain weight test for spring wheat. Studies on the influence of P on wheat gluten composition are relatively scarce, possibly due to existing evidence suggesting that P has only a minor effect on wheat grain composition^73^. Reports by Tóth et al. confirmed that low P treatment had much smaller effects on all protein-related parameters compared to low N treatment^95,96^.

In this study, the gluten index in wheat flour exhibited a range from 22 to 93. According to Curic, et al.^97^, flour with a gluten index in the range of 75 to 90 typically provides good baking quality. Therefore, wheat flour from the minimum tillage treatment received a more favorable rating, with a gluten index ranging from 47 to 93. Research by Šíp, et al.^63^ indicated a wide variation in the gluten index of wheat cultivars, ranging from 65 to 98. Consistent with our study, Buczek, et al.^78^ reported that the gluten index in no-tillage treatment was considerably lower compared to conventional tillage and reduced tillage. Additionally, Haliniarz, et al.^98^ found that CT outperformed no-tillage in terms of grain density and uniformity, resulting in a significantly lower gluten index. A low gluten index suggests the weakness of gluten for the baking industry^99^. Gluten with a low index value has high elasticity but lacks tensile strength, diminishing its gas-holding power during dough formation^99,100^.

The volume of bread stands as a key criterion for assessing the quality of bread-making flour. In this study, the bread volume exhibited a decrease with the intensification of tillage practices, but the use of P fertilizer did not have a significant impact. Notably, the bread volume in our study showed a negative and significant correlation with grain protein percentage and gluten weight (Table 3). Cetiner, et al.^101^ highlighted that very strong flour/dough, such as the Cavus cultivar, renowned as one of the strongest bread-making cultivars in Turkey, may result in loaves with reduced volume and symmetry due to its extremely stiff gluten structure. To address this, gluten proteins in such dough treatments can be modified using reducing agents, diluted with weaker flour, or blended with non-wheat flour to produce bread of higher quality^102,103^.

Moisture content in wheat is a crucial factor influencing its quality, as the dry matter of the grain is dependent on the moisture level. The presence of water absorption in wheat flour is associated with the quantity and quality of protein, damaged starch, and wheat polysaccharides such as beta-glucans and pentosans. This moisture content is particularly significant in the bread-making process^104^. In your study, the moisture percentage of wheat grain after harvest was not affected by tillage and fertilization treatments. However, it's noteworthy that Yousefian, et al.^88^ demonstrated that as the soil tillage level decreases from conventional tillage to no-tillage, the average grain water content increases significantly from 7.0% to 7.57%. It's important to mention that no statistical difference was found for the water content of seeds in CT and reduced tillage treatments in their study.

While the percentage of water absorption was not influenced by tillage and fertilization treatments, the highest amount was observed in the conventional tillage treatment with the use of P fertilizer (64.6%). As highlighted by Garcia del Moral, et al.^105^, an increase in the weight of a thousand grains can lead to a decrease in the percentage of protein per unit volume. This relationship is linked to the milling ability, where a greater weight of a thousand grains results in more flour extraction, subsequently increasing water absorption. In our study, there was a positive and significant correlation between protein percentage and flour water absorption (Table 3). The rise in flour water absorption contributes to the formation of a more regular gluten network, resulting in a more suitable structure before baking bread. Increased water absorption enhances product storage time, improves dough spreadability, reduces moisture loss during baking, and partially enhances bread taste. The absorbed water during baking contributes to a moist texture in fresh bread, and its release during the bread storage period reduces the hardness and brittleness of the resulting bread texture^106^.

## Conclusions

In conclusion, this study highlights the significant impact of tillage practices and phosphorus (P) fertilizer application on various agronomic and quality parameters of wheat. Tillage treatment had a predominant individual effect, significantly influencing most of the measured parameters, including wheat grain and straw yields, yield components, and critical quality attributes such as protein percentage, gluten content, and sedimentation volume. Conventional tillage treatments generally demonstrated favorable outcomes, particularly when combined with P fertilizer application. The application of P fertilizer also played a crucial role, significantly enhancing several yield and quality parameters, regardless of the tillage method employed. Notably, the interaction between tillage and fertilization was highly significant for many of the evaluated characteristics, underscoring the importance of considering their combined effects on wheat productivity and quality. The results suggest that the conventional tillage system combined with P fertilizer application may be the most optimal management practice to achieve the highest wheat grain and straw yields, as well as desirable quality attributes, such as elevated protein content, gluten quality, and sedimentation volume. However, the no-tillage system with P fertilizer also showed promising results in terms of maintaining high grain and straw yields, along with improved bread volume and gluten index. While this study provides valuable insights, it is essential to acknowledge its limitations and the need for further research. Future studies should explore the sustained effects of tillage and fertilization practices over multiple growing seasons, the role of beneficial soil microorganisms, the influence of climate variability, and the economic implications of different management strategies. Comprehensive investigations encompassing crop rotation, soil amendments, and irrigation regimes could further enhance our understanding of sustainable agricultural practices and their impact on wheat quality and productivity.

### Supplementary Information

Supplementary Tables.

## Data Availability

The data used to support the findings of this study are available from the corresponding author upon a reasonable request.
