# Supplementary material for: Impact of varied tillage practices and phosphorus fertilization regimes on wheat yield and grain quality parameters in a five-year corn-wheat rotation system
Source: Sci Rep. 2024 Jun 26;14:14717. doi: 10.1038/s41598-024-65784-w (PMC11208505; doi:10.1038/s41598-024-65784-w)
Supplement: Supplementary file 1 — Supplementary Tables. [file 41598_2024_65784_MOESM1_ESM.docx]

Table S1: Analysis of variance (ANOVA) of effect of various treatments on some measured parameters in this study

|  | | | | | | | | | |
| --- | --- | --- | --- | --- | --- | --- | --- | --- | --- |
| Sources of variance | *df* | Mean square | | | | | | | |
|  |  | Straw N conc. | Straw K conc. | Straw P conc. | Grain N conc. | Grain K conc. | Grain P conc. | Grain yield | Straw yield |
| Repetition (R) | 2 | ^ns^ 0.002 | ^ns^ 0.000 | ^ns^ 0.001 | ^ns^ 0.013 | 0.000006^ns^ | ^ns^ 0.005 | 0.058^ns^ | 0.160 ^ns^ |
| Tillage (T) | 2 | 0.009 ^ns^ | 1.950^**^ | 0.133^**^ | 0.266^*^ | 0.000701^*^ | 0.020^*^ | 3.833^*^ | 21.585^**^ |
| Fertilizer (F) | 1 | ^ns^ 0.002 | 1.135^**^ | 0.009^**^ | ^ns^ 0.035 | 0.000800^*^ | 0.012^*^ | 6.842^**^ | 13.320^**^ |
| F× T | 2 | ^ns^ 0.001 | 1.196^**^ | 0.002^**^ | ^*^ 0.014 | 0.000112^ns^ | 0.005^*^ | ^*^ 0.049 | 1.035^**^ |
| R × T | 4 | ^ns^ 0.001 | ^ns^ 1.39 | 0.000^*^ | ^ns^ 0.017 | 0.000100^ns^ | ^ns^ 0.003 | ^ns^ 0.235 | 0.620^*^ |
| C.V% | | 19.48 | 3.36 | 2.96 | 5.45 | 8.18 | 10.02 | 11.59 | 5.28 |

Respectively; ns, not significant; *significant at *p* < 0.05; **significant at *p* < 0.01

Table S2: Analysis of variance (ANOVA) of effect of various treatments on some measured parameters in this study

|  | | | | | | | |
| --- | --- | --- | --- | --- | --- | --- | --- |
| Sources of variance | df | Mean square | | | | | |
|  |  | Biological yield | 1000 grain weight | No. of spike | Plant height | Spike length | No. of grain per spike |
| Repetition (R) | 2 | ^ns^ 0.161 | ^ns^ 1.608 | ^ns^ 42.667 | ^ns^ 0.129 | 0.011 ^ns^ | ^ns^ 3.500 |
| Tillage (T) | 2 | 38.854^**^ | ^ns^ 1.950 | 22664.000^**^ | 585.629^**^ | 12.3015^**^ | 230.167^**^ |
| Fertilizer (F) | 1 | 30.594^**^ | ^ns^ 0.980 | 28800.000^**^ | 16.037^**^ | ^ns^ 0.125 | 320.889^**^ |
| F× T | 2 | ^**^ 0.420 | ^**^ 7.254 | 5688.000^**^ | 22.705^**^ | ^ns^ 0.476 | 18.056^*^ |
| R × T | 4 | ^ns^ 1.080 | ^ns^ 1.042 | ^ns^ 10.667 | ^ns^ 0.797 | ^ns^ 0.135 | ^ns^ 5.667 |
| C.V% | | 5.45 | 1.46 | 3.25 | 0.66 | 4.71 | 6.11 |

Respectively; ns, not significant; *significant at *p* < 0.05; **significant at *p* < 0.01

Table S3: Effect of various treatments on some measured parameters in this study

| Treatments | Mean | | | | | | | |
| --- | --- | --- | --- | --- | --- | --- | --- | --- |
|  | Straw N conc. | Straw K conc. | Straw P conc. | Grain N conc. | Grain K conc. | grain P conc. | Grain yield | Straw yield |
|  | % | % | % | % | % | % | t ha^-1^ | t ha^-1^ |
| **Tillage** |  | | | | | | | |
| CT | 0.28^ab^ | 0.60^b^ | 0.07^c^ | 2.05^a^ | 0.118^a^ | 0.20^b^ | 4.13^a^ | 7.56^a^ |
| MT | 0.33^a^ | 1.47^a^ | 0.17^b^ | 1.91^a^ | 0.108^ab^ | 0.28^ab^ | 3.75^a^ | 8.23^a^ |
| NT | 0.25^b^ | 0.40^c^ | 0.37^a^ | 1.63^b^ | 0.097^b^ | 0.31^a^ | 2.59^b^ | 4.66^b^ |
| Mean | 0.29 | 0.82 | 0.20 | 1.86 | 0.109 | 0.26 | 10.47 | 6.82 |
| **Fertilizer** |  | | | | | | | |
| P0 | 0.30^a^ | 1.07^a^ | 0.23^a^ | 1.91^a^ | 0.10^b^ | 0.23^b^ | 2.87^b^ | 5.96^b^ |
| RP | 0.28^a^ | 0.57^b^ | 0.18^b^ | 1.82^a^ | 0.11^a^ | 0.29^a^ | 4.11^a^ | 7.67^a^ |
| Mean | 0.29 | 0.82 | 0.21 | 1.87 | 0.105 | 0.26 | 3.49 | 6.82 |

CT, conventional tillage; MT, minimum tillage; NT, no tillage; P0, no phosphorus fertilizer use; PR, 100% fertilizer recommendation. Different letters indicate significant differences according to the Duncan at *p*≤ 0.05

Table S4: Effect of various treatments on some measured parameters in this study

| Treatments | Mean | | | | | |
| --- | --- | --- | --- | --- | --- | --- |
|  | Biological yield | 1000 grain weight | No. of spike | Plant height | Spike length | No. of grain per spike |
|  | t ha^-1^ | g | In m^2^ | cm | cm |  |
| **Tillage** |  | | | | |  |
| CT | 12.43^a^ | 46.83^a^ | 506.00^a^ | 94.67^a^ | 10.56^a^ | 41.17^a^ |
| MT | 10.25^b^ | 47.29^a^ | 432.00^b^ | 83.17^b^ | 9.67^b^ | 40.00^b^ |
| NT | 7.35^c^ | 46.16^a^ | 384.00^c^ | 75.00^c^ | 7.75^c^ | 34.83^c^ |
| Mean | 10.01 | 46.76 | 440.67 | 84.28 | 9.33 | 38.67 |
| **Fertilizer** |  | | | | |  |
| P0 | 8.71^b^ | 46.53^a^ | 400.67^b^ | 83.33^b^ | 9.24^a^ | 36.44^b^ |
| RP | 11.31^a^ | 46.99^a^ | 480.67^a^ | 85.22^a^ | 9.41^a^ | 44.89^a^ |
| Mean | 10.01 | 46.76 | 440.67 | 84.28 | 9.33 | 40.67 |

CT, conventional tillage; MT, minimum tillage; NT, no tillage; P0, no phosphorus fertilizer use; PR, 100% fertilizer recommendation. Different letters indicate significant differences according to the Duncan at *p*≤ 0.05

Table S5: ANOVA of effect of various treatments on some measured quality parameters of grains in this study

| Sources of variance | df | Mean square | | | | | | | |
| --- | --- | --- | --- | --- | --- | --- | --- | --- | --- |
|  |  | Protein content | Zeleny sedimentation volume | Bread Volume | Moisture content | Hardness index | Flour water absorption | Wet gluten | Gluten index |
| Repetition (R) | 2 | ^ns^ 0.02 | ^ns^ 0.22 | ^ns^ 1085.39 | ^ns^ 0.35 | 3.56^*^ | ^ns^ 1.05 | 1.50^ns^ | 40.17^ns^ |
| Tillage (T) | 2 | 1.09^**^ | ^*^ 6.06 | 1793.39^*^ | ^ns^ 0.67 | 2.72^*^ | ^*^ 0.98 | 95.17^**^ | 2108.17^**^ |
| Fertilizer (F) | 1 | ^ns^ 0.04 | ^ns^ 4.50 | 242.00^ns^ | ^ns^ 0.00 | ^ns^ 4.50 | 0.14^ns^ | 53.39^**^ | 1136.06^**^ |
| F× T | 2 | ^ns^ 0.17 | ^ns^ 1.17 | 1915.17^*^ | ^ns^ 1.26 | ^ns^ 2.17 | ^*^1.15 | ^**^ 21.06 | 1124.06^**^ |
| R × T | 4 | ^ns^ 0.06 | ^ns^ 1.39 | ^ns^ 188.56 | ^ns^ 0.45 | ^ns^ 0.31 | ^ns^ 0.31 | 52.39^*^ | 14.08^ns^ |
| C.V% | | 2.21 | 7.54 | 2.69 | 13.65 | 2.19 | 0.60 | 8.56 | 7.30 |

Respectively; ns, not significant; *significant at *p* < 0.05; **significant at *p* < 0.01

Table S6: Effect of various treatments on some measured quality parameters of grains in this study

| Treatments | Mean | | | | | | | |
| --- | --- | --- | --- | --- | --- | --- | --- | --- |
|  | Protein content | Zeleny sedimentation volume | Bread volume | Moisture content | Hardness index | Water absorption | Wet gluten | Gluten index |
|  | (%) | (mL) | (mL) | (%) |  | (%) | (%) |  |
| Tillage |  | | | | |  |  |  |
| CT | 12.00^a^ | 20.33^a^ | 618.17^b^ | 6.38^a^ | 45.00^a^ | 64.12^a^ | 25.83^a^ | 47.00^b^ |
| MT | 11.52^b^ | 19.17^ab^ | 647.33^a^ | 6.67^a^ | 43.67^b^ | 63.33^a^ | 18.00^b^ | 69.83^a^ |
| NT | 11.15^b^ | 18.33^b^ | 648.83^a^ | 6.00^a^ | 44.50^ab^ | 63.55^a^ | 20.67^b^ | 32.67^c^ |
| Mean | 11.56 | 19.28 | 638.11 | 6.35 | 44.39 | 63.67 | 21.50 | 49.83 |
| Fertilizer |  | | | | |  |  |  |
| P0 | 11.51^a^ | 18.78^a^ | 641.78^a^ | 6.36^a^ | 43.89^a^ | 63.58^a^ | 19.78^b^ | 57.78^a^ |
| RP | 11.60^a^ | 19.78^a^ | 634.44^a^ | 6.34^a^ | 44.89^a^ | 63.76^a^ | 23.22^a^ | 41.89^b^ |
| Mean | 11.56 | 19.28 | 638.11 | 6.35 | 44.39 | 63.67 | 21.50 | 49.84 |

CT, conventional tillage; MT, minimum tillage; NT, no tillage; P0, no phosphorus fertilizer use; PR, 100% fertilizer recommendation. Different letters indicate significant differences according to the Duncan at p≤ 0.05
